# Dopamine Receptors in Human Adipocytes: Expression and Functions

**DOI:** 10.1371/journal.pone.0025537

**Published:** 2011-09-26

**Authors:** Dana C. Borcherding, Eric R. Hugo, Gila Idelman, Anuradha De Silva, Nathan W. Richtand, Jean Loftus, Nira Ben-Jonathan

**Affiliations:** 1 Department of Cancer and Cell Biology, University of Cincinnati, Cincinnati, Ohio, United States of America; 2 The Christ Hospital, Cincinnati, Ohio, United States of America; German Institute for Human Nutrition, Germany

## Abstract

**Introduction:**

Dopamine (DA) binds to five receptors (DAR), classified by their ability to increase (D1R-like) or decrease (D2R-like) cAMP. In humans, most DA circulates as dopamine sulfate (DA-S), which can be de-conjugated to bioactive DA by arylsulfatase A (ARSA). The objective was to examine expression of DAR and ARSA in human adipose tissue and determine whether DA regulates prolactin (PRL) and adipokine expression and release.

**Methods:**

DAR were analyzed by RT-PCR and Western blotting in explants, primary adipocytes and two human adipocyte cell lines, LS14 and SW872. ARSA expression and activity were determined by qPCR and enzymatic assay. PRL expression and release were determined by luciferase reporter and Nb2 bioassay. Analysis of cAMP, cGMP, leptin, adiponectin and interleukin 6 (IL-6) was done by ELISA. Activation of MAPK and PI3 kinase/Akt was determined by Western blotting.

**Results:**

DAR are variably expressed at the mRNA and protein levels in adipose tissue and adipocytes during adipogenesis. ARSA activity in adipocyte increases after differentiation. DA at nM concentrations suppresses cAMP, stimulates cGMP, and activates MAPK in adipocytes. Acting via D2R-like receptors, DA and DA-S inhibit PRL gene expression and release. Acting via D1R/D5R receptors, DA suppresses leptin and stimulates adiponectin and IL-6 release.

**Conclusions:**

This is the first report that human adipocytes express functional DAR and ARSA, suggesting a regulatory role for peripheral DA in adipose functions. We speculate that the propensity of some DAR-activating antipsychotics to increase weight and alter metabolic homeostasis is due, in part, to their direct action on adipose tissue.

## Introduction

Catecholamines (CA) are synthesized from tyrosine by a sequential enzymatic conversion of dopamine (DA) to norepinephrine (NE) and epinephrine (EPI). DA binds to five G-protein-coupled, seven transmembrane domain receptors (DAR), classified as those that are linked to stimulation (D1R and D5R) or inhibition (D2R, D3R and D4R) of adenylate cyclase (AC) [1], [2]. When coupled to Gαs proteins, D1-like receptors can activate the protein kinase A (PKA), mitogen activated protein kinase (MAPK), and cGMP/PKG pathways [3]–[6]. The D2-like receptors are coupled to Gαi/o proteins and inhibit AC, followed by the suppression of cAMP [7]. DA is a pleiotropic compound that acts as a neurotransmitter and a hormone. In the brain, DA rapidly alters electrical activity, ion channels and neurotransmitter release. In peripheral non-neuronal tissues, e.g., pituitary, kidney and blood vessels, DA acts more slowly and affects electrolyte transport, vasodilation, hormone production and cell proliferation [7], [8].

DA is the primary inhibitor of pituitary prolactin (PRL) release [9]. Unique to humans, PRL is also produced in multiple extrapituitary sites, where it functions as a cytokine [10]. After discovering *de novo* synthesis of PRL in human adipose tissue [11], [12], we examined its regulation. When adipocytes were placed in culture, PRL release increased for several days [13]. This resembled the progressive rise in PRL release from cultured pituitary cells, which is attributable to the removal of tonic inhibition by hypothalamic DA [14]. We initially ruled out DA as the inhibitor of adipose PRL because a ready source of DA to the adipocytes was not apparent, and there was no information on DAR expression in human adipose tissue, except for a single report describing a novel DAR in rat brown adipose tissue [15].

Dopamine sulfate (DA-S) is the major form of circulating DA in humans [16], [17]. Sulfoconjugation is carried out in the gastrointestinal (GI) tract by SULT1A3 sulfotransferase [16], [18]. Basal serum DA-S levels at ≈10 nM exceeds by 5 fold the combined levels of free DA (1.5 nM), NE (1 nM) or Epi (0.2 nM). The biologically inactive DA-S has a serum half-life of 3–4 hr, compared to several minutes for unmodified DA [19]. However, unlike inactivation of DA by deamination, O-methylation or glucuronidation, sulfoconjugation is reversible, and DA-S can be converted back to bioactive DA by arylsulfatase A (ARSA), a releasable lysosomal enzyme [20], [21]. We reasoned that if adipocytes express DAR and possess an active ARSA, circulating DA-S can serve as an inhibitor of adipose PRL.

Production of adipokines is a major function of adipose tissue. Leptin is an important adipokine which is involved in body weight regulation via its effects on food intake and energy expenditure [22]. Adiponectin is a key adipose-derived hormone which plays critical roles in fuel homeostasis, insulin action and atherosclerosis [23]. Interleukin-6 (IL-6) is an inflammatory cytokine which is produced by both macrophages and adipocytes and whose release is increased in obesity [24]. The production/release of these adipokines is differentially regulated in human adipocytes by beta adrenergic receptor (β-AR) agonists [25]–[28]. DA was recently reported to suppress leptin expression and release in 3T3-L1 murine adipocytes [29], but there is no information on comparable effects of DA on human adipocytes.

The objective of this study was to detect expression of DAR in human adipocytes at both the mRNA and protein levels and confirm their functionality by examining the effects of DA on PRL, leptin, adiponectin and IL-6 release. To this end, we undertook a comprehensive approach and used adipose tissue explants, primary adipocytes and two human adipocyte cell lines: LS14 which we have cloned from a metastatic liposarcoma [30], and SW872, a liposarcoma-derived cell line obtained from the ATCC. Both cell lines differ with respect to gene expression and the timing of full differentiation.

## Materials and Methods

### Ethics Statement

Written informed consents, approved by the Institutional Review Board of the Christ Hospital of Cincinnati, were obtained from all participants.

### Patients, explant preparation and cell harvesting

Subcutaneous (sc) adipose tissue was obtained from non-obese, non-diabetic women (BMI<30) undergoing elective abdominoplasty. Adipose explants were placed in 48-well plates (80–100 mg/well) containing DMEM:F12 with 1% ITS^+^ (BD Biosciences, San Jose, CA) and incubated with the various treatments. For cell harvesting, tissue fragments were digested with collagenase as described [13]. After filtration and brief centrifugation, the floating mature adipocytes (100 µl of packed cells) were incubated in collagen-coated plates containing DMEM:F12 with 1% ITS^+^ and the various treatments. The stromal vascular cell (SVC) fraction which contains the preadipocytes was sedimented, and after treatment with erythrocyte lysis buffer, cells were cultured in DMEM:F12 with 10% FBS and 50 µg/ml Primocin (Invitrogen, San Diego, CA). After 2–3 passages, cells were frozen until used.

### Cell culture and differentiation

LS14 cells were cultured as described [13], while SW872 cells were maintained in DMEM:F12 with 10% FBS. For differentiation, primary sc preadipocytes, SW872 and LS14 cells were plated in collagen-coated 24 well plates at 100,000 cells/well. Cells were incubated in serum-free basal adipogenesis medium (BAM) as described [13]. Adipogenesis was induced by the addition of 250 µM IBMX (BioMol, Plymouth Meeting, PA). After three days, cells were incubated in BAM without IBMX, and cell differentiation was monitored visually. On days 10–14, 80–90% of either primary or cell lines, showed lipid accumulation. After overnight incubation in DMEM/F12 plus 1% CSS, cells were treated as described below.

### Generation of stably transfected SW872 cells and luciferase assay

The full-length superdistal PRL promoter (dPRL) cloned into the PGL3E luciferase reporter vector (Promega, San Louis Obispo, CA) was provided by Dr. Brar, Children's Hospital, Cincinnati, OH. SW872 cells were co-transfected with dsRed2-N1 plasmid (Clontech, Mountain View, CA) and the dPRL/PGL3E construct at a 1∶10 ratio using Fugene 6 (Roche, Indianapolis, IN). After 72 hr, cells were selected using 0.8 µg/ml G418 (Sigma, St Louis, MO). Colonies were picked, expanded and the cells were maintained thereafter in 0.08 µg/ml G418. The stably transfected cells were induced to differentiate, serum starved, and treated as indicated below. After lysing with Passive Lysis Buffer (Promega), luciferase activity was determined using Microplate Luminometer (Dynex Technologies, Chantilly, VA).

### Conventional and Real-time RT-PCR

RNA isolation and cDNA synthesis were done as described [13]. Conventional PCR was performed using intron-spanning primers (Table 1). For D1R/D5R which have no introns, DNAse was added during RNA isolation. Additionally, samples were evaluated for genomic DNA contamination using no reverse transcriptase control. Cycle conditions were: 96 C for 6 min for polymerase activation, followed by 35 cycles of 94, 57, and 72 C, each for 45 sec. Products were resolved on a 1.5% agarose gel containing ethidium bromide and photographed. Quantitative real-time PCR (qPCR) was performed with SYBR Green and the appropriate primers (Table 1). Fluorometric products were detected with Applied Biosystem StepOnePlus instrument. Cycle parameters were: 96 C for 6 min, followed by 45 cycles of 94 C for 15 sec, 57 C for 15 sec, and 72 C for 30 sec, and an optical read at 83.5 C for 6 sec. Product purity was confirmed by DNA melting curve analysis and agarose gel electrophoresis. PCR efficiency was determined using the LinRegPCR program or by cDNA dose response curve analysis. β2-Microglobulin (B2M) was used as a reference gene. Fold changes in gene expression were calculated from cycle threshold and efficiency measurements as described [13].

**Table 1:** Gene-specific PCR primers for conventional and quantitative real time PCR.

Gene	Genbank Accession Number	Forward primer (5′→3′)	Reverse Primer (5′→3′)	AmplimerSize (bp)
B2M	NM_004048	GGCATTCCTGAAGCTGAC	GAATCTTTGGAGTACGCTGG	114
ARSA	NM_000487	TATGCCTCTCACCACAC	GGTCTCAGGTCCATTGTC	191
ARSB	NM_000046	GCTACCAGATCCGTACAG	TTCCGGTACATTCCCAG	153
ARSC	NM_000351	AGCACTGATAGGGAAATGG	AGCAAGGGTAAGGAGGG	216
DRD1	NM_000794	CTCCGTTTCCAAATACATTCCA	CACTGTTGATTCTTTGCCCT	169
DRD2	NM_000795	AGCATCGACAGGTACACAG	CTCGTTCTGGTCTGCGT	159
DRD3	NM_033660	GTGGTATACCTGGAGGTGAC	GCAGTGTACCTGTCTATGCT	136
DRD4	NM_000797	CCGCTCTTCGTCTACTC	ACAGGTTGAAGATGGAGG	114
DRD5	NM_000798	CTCATCTCCTACAACCAAGAC	TGATAGATCTGGAACATGCGA	148
PRL	NM_000948	TTCAGCGAATTCGATAAACGG	TGATACAGAGGCTCATTCCAG	181
PRLR	NM_000949	CGTGACTTACATAGTTCAGCCA	GGAGCGTGAACCAACCA	139

All primer sets were intron-spanning except those for D_1_ and D_5_ dopamine receptors. B2M-β_2_ microglobulin, ARSA-arylsulfatase A, ARSB-arylsulfatase B, ARSC-arylsulfatase C (steroylsulfatase), DRD1-D_1_ dopamine receptor, DRD2-D_2_ dopamine receptor, DRD3-D_3_ dopamine receptor, DRD4-D_4_ dopamine receptor, DRD5-D_5_ dopamine receptor, PRL-prolactin, PRLR-prolactin receptor.

### PRL release studies

Adipose tissue explants, mature adipocytes or differentiated primary adipocytes, LS14 and SW872 cells were incubated with DA, bromocriptine (a D2R agonist), isoproterenol (all from Sigma), dopamine-4-O-sulfate (from NIMH) or raclopride, a D2R antagonist (Tocris, Ellisvile, MO). After 24 h, conditioned media (CM) were collected and aliquots were analyzed for PRL by the Nb2 bioassay, which is approximately 50 times more sensitive than RIA or ELISA for PRL [13]. Briefly, rat Nb2 lymphocytes were serum-starved and plated in 96-well plates at 30,000 cells/well. Cells were incubated with recombinant human PRL (Protein Laboratories, Rehovot, Israel) in triplicate or with CM aliquots in duplicate. After 72 h, cell viability was determined by fluorescence using resazurin reduction. PRL concentration in CM was calculated from a standard curve, with the lowest detectable level of 2 pg/well. Nb2 cell proliferation was not affected by the tested compounds.

### Western blot analysis

Cells were homogenized in lysis buffer and 35 µg of lysate proteins were separated on 12% SDS gels and transferred to nitrocellulose membranes. For DAR, validated antibodies [31]–[33] against D1R (Calbiochem, San Diego, CA: 324390; 1∶2,000), D2R (Santa Cruz, CA: SC-5303; 1∶250), and D4R (Calbiochem: 324405; 1∶1000) were used. For signaling pathways, ERK1/2 (ab #9102) phospho ERK 1/2 (Thr202/Tyr204 ab #9101S), Phospho Akt (Ser473, ab #9271S) and Akt (ab #9272), all from Cell signaling (Danvers, MA) were used at 1∶1000. After incubation with horseradish peroxidase-conjugated secondary antibodies, products were exposed to SuperSignal chemiluminescence reagents (Pierce, Rockford, IL) and photographed; β-actin (Sigma, A1978;1∶5000) was used as a loading control.

### Arylsulfatase A Activity

ARSA activity was determined by the method of Chang et al [34] after modifications. Briefly, samples were homogenized in 0.05 M acetate buffer, pH 5.0, freeze-thawed, and centrifuged at 12,000× g for 5 min. Lysates (15 µg) or CM (X10 concentrated) were incubated at 37C in 0.05 M acetate buffer, pH 5.6 containing 3 mM lead acetate, and 5 mM 4-methylumbelliferyl sulfate (Sigma), with or without 3 mM Ag+. After 30 min, the reaction was stopped with 0.2 M glycine-carbonate buffer, pH 10.4 and 1 mM EDTA. Fluorescence was measured at 370 nm excitation and 450 nm emission, using Gemini fluorescent microplate reader (Molecular Devices, Sunnyvale, CA). Enzyme activity was calculated from a standard curve.

### cAMP and cGMP determinations

Cells were incubated with the various treatments for 30 min and then lysed in 0.1 M HCl. After centrifugation, the supernatant was analyzed for cAMP and cGMP using respective ELISA kits from Cayman Chemical Co (Ann Arbor, MI). To increase assay sensitivity for cGMP analysis, samples were first acetylated according to manufacturer's instructions.

### Adipokine determination

Fluorescent sandwich ELISAs for human leptin, adiponectin and IL-6 were optimized in our lab as described [35]. Briefly, matched monoclonal ab pairs against leptin (R&D Systems, Minneapolis MN; MAB398 for capture and BAM398 for detection), adiponectin (R&D systems; MAB10651 for capture and BAM1065 for detection) and IL-6 (Invitrogen, Carlsbad CA; AHC0562 for capture and AHC0469 for detection) were used. Plates coated with the capture ab were co-incubated with biotinylated detection ab, antigen standards and CM from explants or cells. Streptavidin-conjugated horseradish peroxidase was added, followed by a fluorimetric substrate (QuantaBlue; Thermo-Fisher, Rockford, IL). Plates were read at 325 nm excitation and 420 nm emission.

### Data Analysis

Experiments were repeated at least 3 times. Values were expressed as means±SEM. Data were analyzed by Student's t test or ANOVA. P<0.05 was considered significant.

## Results

### Expression of DAR in adipose tissue and adipocytes

**Figure 1:**
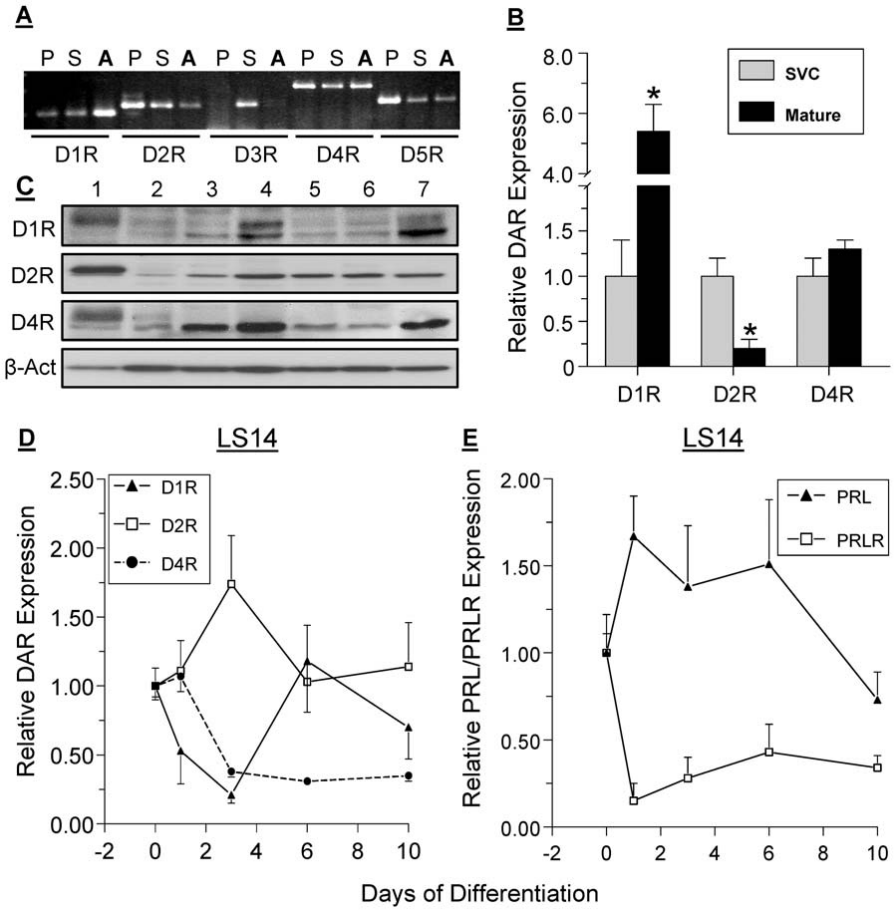
DAR expression in human adipose tissue and adipocytes. **A**, comparison of DAR expression in human pituitary (P), striatum (S) and sc adipose tissue (A), as determined by conventional RT-PCR. **B**, expression of selected DAR in the stromo-vascular cell (SVC) fraction and mature adipocytes, as determined by real-time PCR. Data are expressed as relative changes over SVC. Each value is a mean±SEM of 3 determinations; *, p<0.05. **C**, Immunoblot of selected DAR proteins in tissues and cells. Lanes: 1, pituitary; 2, proliferating primary preadipocytes; 3, proliferating LS14; 4, proliferating SW872; 5, differentiated primary adipocytes; 6, differentiated LS14; 7, differentiated SW872. Each lane was loaded with 40 µg proteins, except for the pituitary (30 µg proteins). β-actin (β-Act) was used as a loading control. Expression of D1R, D2R and D4R (**panel D**), and PRL vs PRLR (**panel E**) during adipogenesis in LS14 cells was determined by qPCR. Data are expressed as relative changes over day 0, and were calculated from the cycle threshold and efficiency measurements (Mean±SEM of 3 determinations).

Conventional RT-PCR was used to compare the expression of DAR in sc adipose tissue, striatum and pituitary. Four of the five DAR were detected in adipose tissue, with D1R being the most abundant (**Fig. 1A**). As expected, the pituitary showed high expression of D2R, D4R and D5R, while the striatum showed differential expression of all DAR. As determined by qPCR, the relative expression of D1R is higher, while that of D2R is lower in mature adipocytes than the SVC fraction, while expression of D4R was similar in the two fractions (**Fig. 1B**).

Western blotting was used to compare expression of the DAR proteins in primary adipocytes, LS14 and SW872 cells before and after differentiation. **Fig. 1C** shows that D1R, D2R and D4R proteins were expressed at variable amounts in the pituitary (serving as a positive control), and in the three types of adipocytes. There was an apparent downregulation of D1R and D4R, but upregulation of D2R, in LS14 cells after differentiation. All receptors examined showed some evidence of isoforms.

Changes in D1R, D2R and D4R mRNA levels during adipogenesis in LS14 cells were determined by qPCR. **Fig. 1D** shows a reduction in D1R, but an increase in D2R, during the first three days of differentiation. Expression of D4R was reduced on day 3 and remained suppressed until day 10. PRL expression was increased during the first six days of differentiation, followed by a decline, whereas PRLR expression showed a significant reduction throughout adipogenesis (**Fig. 1D**).

### Adipocytes express an active ARSA and respond to DA-S

**Figure 2:**
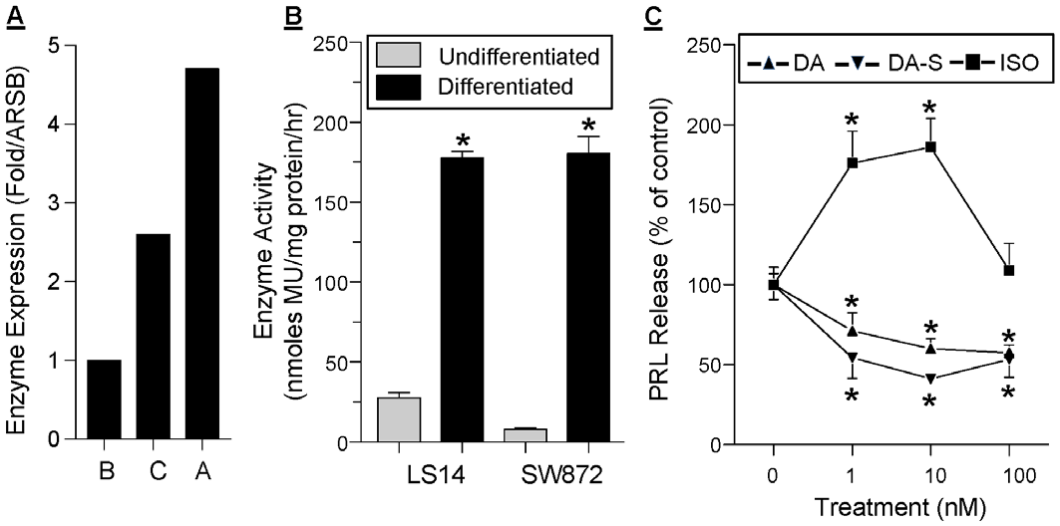
Expression of active arylsulfatase A (ARSA) in adipose tissue and adipocytes and confirmation of DA-S bioactivity. **A**, comparison of mRNA levels of ARSA (A), ARSB (B) and ARSC (C) in sc adipose tissue, as determine by qPCR. Data are expressed as fold changes in gene expression over ARSB, and were calculated from the cycle threshold and efficiency measurements. **B**, ARSA activity before (light bars) and after (dark bars) differentiation of LS14 and SW872 adipocytes. Specific ARSA activity was determined by subtracting enzyme activity in the presence of silver nitrate from total enzyme activity. Each value is a mean±SEM of 4 determinations; *, p<0.05. **C**, both DA and DA-S inhibit PRL release from sc adipose tissue explants, whereas isoproterenol (ISO), a β-AR agonist, causes stimulation. Explants were incubated with the different compounds for 24 hr and PRL concentration in CM was determined by the Nb2 bioassay. Data are expressed as % of control. Each value is a mean±SEM of 6 determinations; *, p<0.05.

We used qPCR to compare the expression of arylsulfatase A (ARSA), specific for catecholamines and cerebrosides, arylsulfatase B (ARSB), specific for glycosaminoglycans, and arylsulfatase C (ARSC), specific for steroids [21]. The mRNA levels of ARSA and ARSC in sc adipose tissue were 4.5 and 2.5 fold higher, respectively, than those of ARSB (**Fig. 2A**). An enzymatic assay that measures total arylsulfatase activity was then employed. In the presence of silver nitrate, only ARSA activity is blocked, enabling the calculation of its activity by subtraction from total enzyme activity. As shown in **Fig. 2B**, basal ARSA activity was detectable in non-differentiated LS14 and SW872 cells, increasing 8 and 20 fold, respectively, after differentiation. ARSA activity was also detectable in CM from differentiated adipocytes (data not shown). To determine whether adipocytes can convert DA-S to bioactive DA, the effects of DA and DA-S on PRL release from sc adipose explants were compared. **Fig. 2C** shows a similar inhibition of PRL release by both DA and DA-S. The β-AR agonist isoproterenol (ISO), stimulated PRL release, showing an inverted U-shaped dose-dependent curve.

### PRL release from all types of adipocytes is inhibited by both DA and bromocriptine

**Figure 3:**
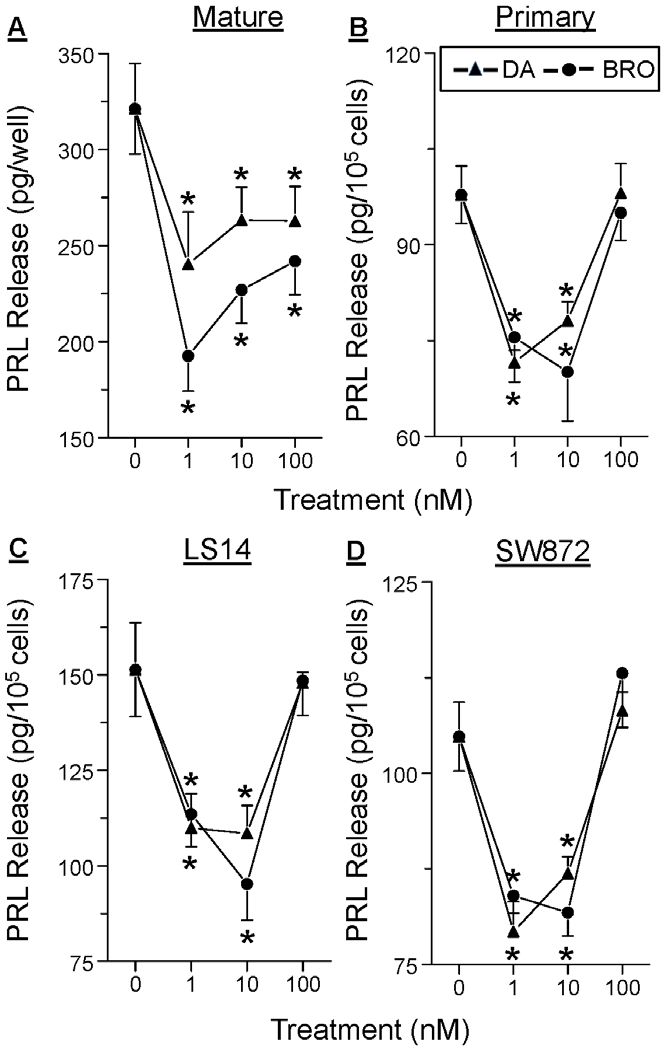
DA and bromocriptine (BRO) inhibit PRL release from different types of adipocytes. **A**, isolated sc mature adipocytes. **B**, differentiated sc primary adipocytes. **C**, differentiated LS14 cells. **D**, differentiated SW872 cells. In each case, cells were incubated with different doses of DA or bromocriptine (BRO) for 24 hr, and PRL in CM was determined by the Nb2 bioassay. Each value is a mean±SEM of 6 determinations; *, p<0.05.

We next examined whether DA directly affects PRL release by the adipocytes. **Fig. 3** shows a similar DA-induced inhibition of PRL release from mature adipocytes (**panel A**), differentiated primary preadipocytes (**panel B**), differentiated LS14 cells (**panel C**), and differentiated SW872 cells (**panel D**). Bromocriptine (BRO), a specific D2R agonist, mimicked the inhibitory effect of DA. A non-monotonic dose-dependent inhibition of PRL release was apparent in all cases, with 1 and 10 nM, but not 100 nM, of DA and BRO showing effective inhibition.

### Both DA and DA-S suppress PRL gene expression via the superdistal promoter

**Figure 4:**
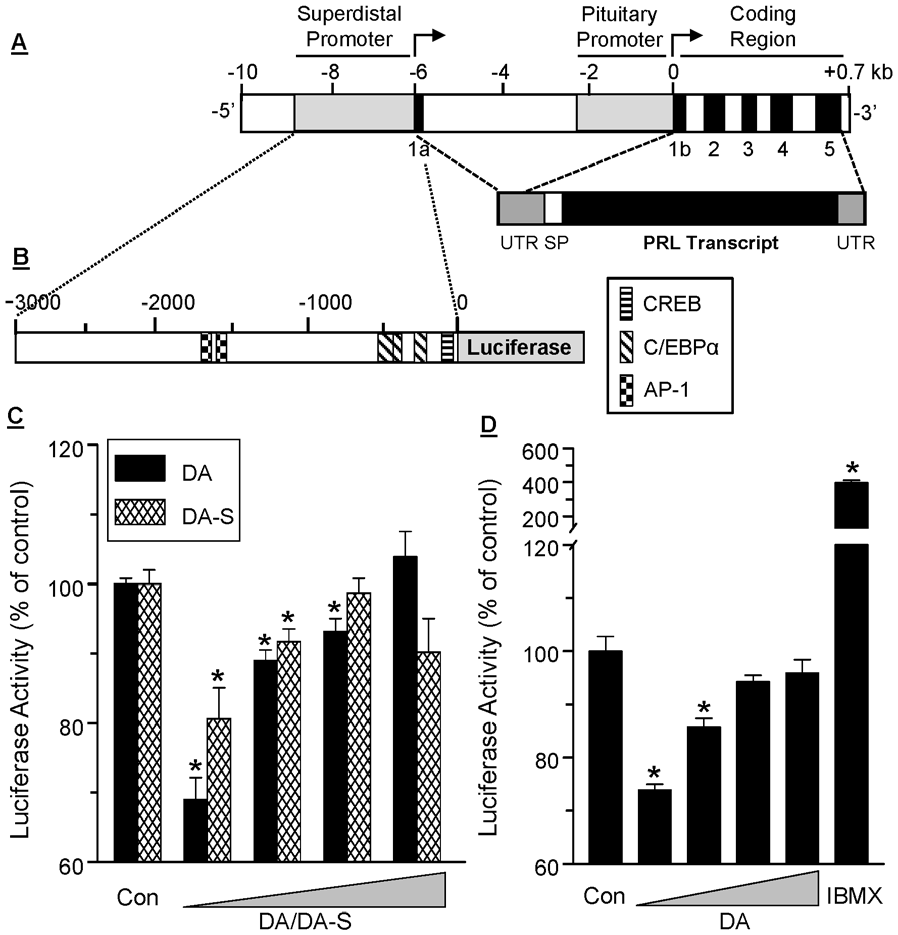
Both DA and DA-S suppress adipocyte PRL gene expression via the superdistal promoter. **A**, diagram of the pituitary and superdistal promoters which regulate pituitary and extrapituitary PRL expression, respectively; both generate an identical PRL protein. UTR, untranslated region; SP, signal peptide. **B**, the superdistal PRL promoter construct driving a luciferase reporter that was stably transfected in SW872 cells. Also shown are putative transcription factor binding sites capable of responding to activation of PKA and MAPK signaling. **C**, inhibition of PRL gene expression by DA and DA-S after 6 hr of incubation; **D**, inhibition of PRL gene expression by DA and stimulation by IBMX after 24 hrs of incubation. Stably transfected SW872 cells were induced to differentiate and then incubated with increasing doses of DA or DA-S (0.1 nM to 100 nM) or with 250 µM IBMX. Luciferase activity was determined in cell lysates by luminommetry. Each value is a mean±SEM of 6 determinations; *, p<0.05.

A diagram of the proximal and superdistal promoters that regulate pituitary and extrapituitary PRL, respectively, is shown in **Fig. 4A**. The superdistal promoter is located 5.8 kB upstream of the pituitary start site [10], and contains putative CREB (cAMP response element binding protein) and c/EBP (CCAAT/enhancer binding protein) transcription binding sites in the proximal region, as well as two AP-1 sites in the more distal region (**Fig. 4B**). SW872 cells, stably transfected with a luciferase reporter driven by the 3000 kb superdistal promoter (**Fig. 4B**), were used. Within 6 hr, as little as 0.1 nM of DA or DA-S suppressed PRL expression (**Fig. 4C**), an effect that was also seen after 24 hr (**Fig. 4D**). As was the case with PRL release (**Fig. 3**), a non-linear, dose-dependent curve was evident, suggesting activation of stimulatory DAR at the higher DA-S/DA doses. IBMX, a phosphodiesterase inhibitor, increased PRL expression 4 fold, supporting the role of the cAMP system in the transcriptional control of adipocyte PRL.

### DAR signal through multiple pathways in the adipocytes

**Figure 5:**
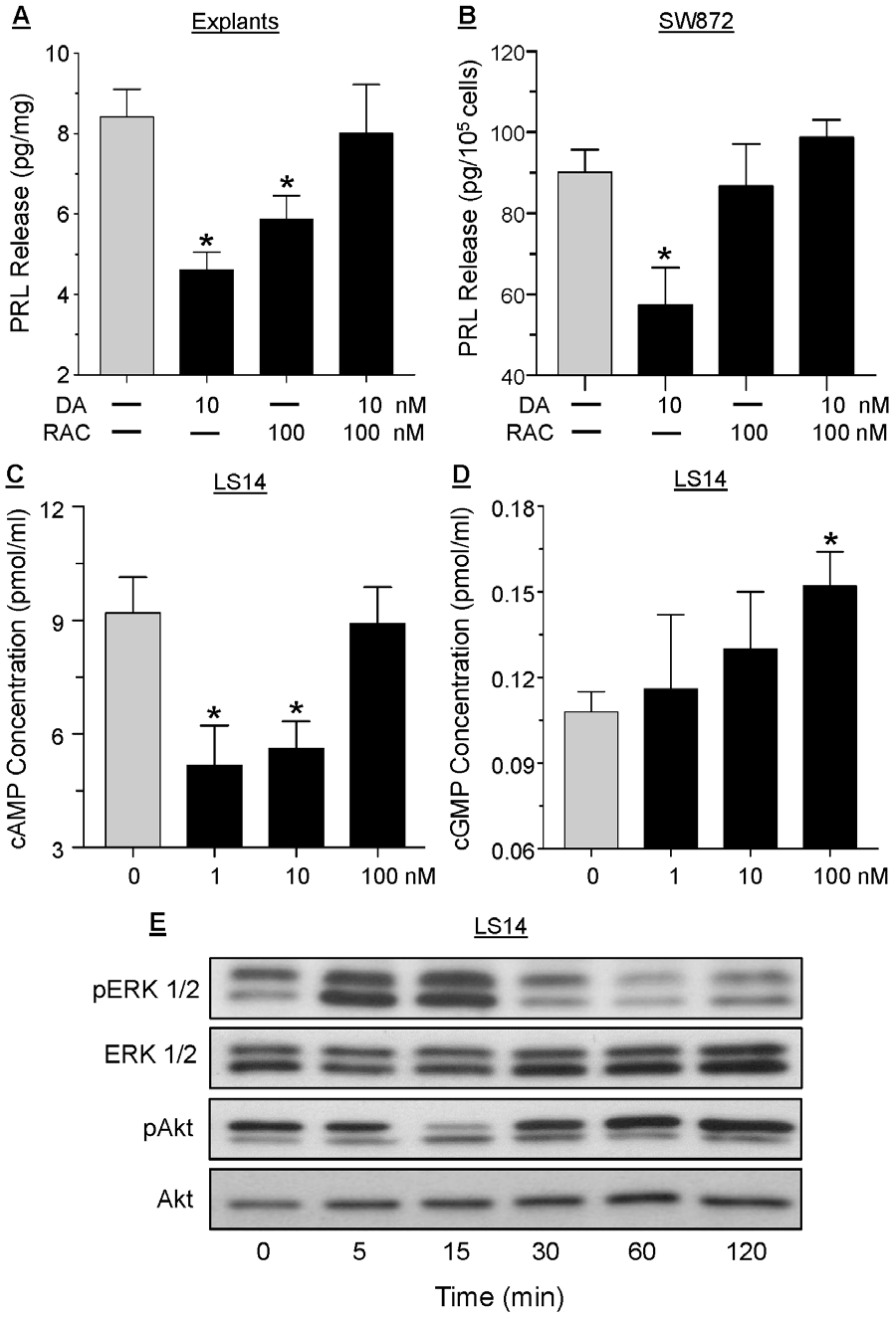
Different signaling pathways mediate DAR actions in the adipocytes. Sc adipose explants (**panel A**), or differentiated SW872 cells (**panel B**) were incubated with 10 nM DA or 100 nM raclopride (RAC), a D2R antagonist, for 24 hr, or pre-incubated with RAC 1 hr before DA addition. PRL in CM was determined by the Nb2 bioassay. Each value is a mean±SEM of 6 determinations; *, p<0.05. Differentiated LS14 cells were incubated with 1, 10 or 100 nM DA for 30 min and intracellular cAMP (**panel C**) or cGMP (**panel D**) levels were determined by respective ELISAs. Each value is a mean±SEM of 4 determinations; *, p<0.05. **E**, differentiated LS14 cells were incubated with 10 nM DA, and cell lysates obtained at different times were analyzed for phosphorylate ERK1/2 (pERK1/2) and phosphorylated Akt (pAkt) by Western blotting. Total ERK1/2 and Akt served as loading controls.

To further characterize the DAR that mediates the suppression of PRL by DA, adipose explants (**Fig. 5A**) and differentiated SW872 cells (**Fig. 5B**) were pretreated with 100 nM raclopride, a selective D2R antagonist, before incubation with 10 nM DA for 24 hrs. In both cases, raclopride abrogated the inhibitory action of DA on PRL release, indicating mediation by D2R. The ability of DA to affect intracellular cAMP and cGMP concentrations in LS14 cells was then examined. Within 30 min of incubation, 1 and 10 nM DA suppressed, while 100 nM DA had no effect, on intracellular cAMP levels (**Fig. 5C**). On the other hand, DA at 100 nM, but not at lower doses, increased cGMP accumulation within 30 min of incubation (**Fig. 5D**); a stimulatory effect on cGMP was also obtained in response to 10 nM ISO (data not shown).

Western blotting was then used to examine the effects of DA on the MAPK and PI3K pathways. As show in **Fig. 5E**, within 5 min of incubating differentiated LS14 cells with 10 nM DA, ERK1/2 phosphorylation increased, remained elevated for another 10 min, and reduced to basal levels by 120 min. Akt phosphorylation was suppressed at 15 min, and was unchanged or slightly stimulated thereafter.

### DA differentially affect the release of selected adipokines

**Figure 6:**
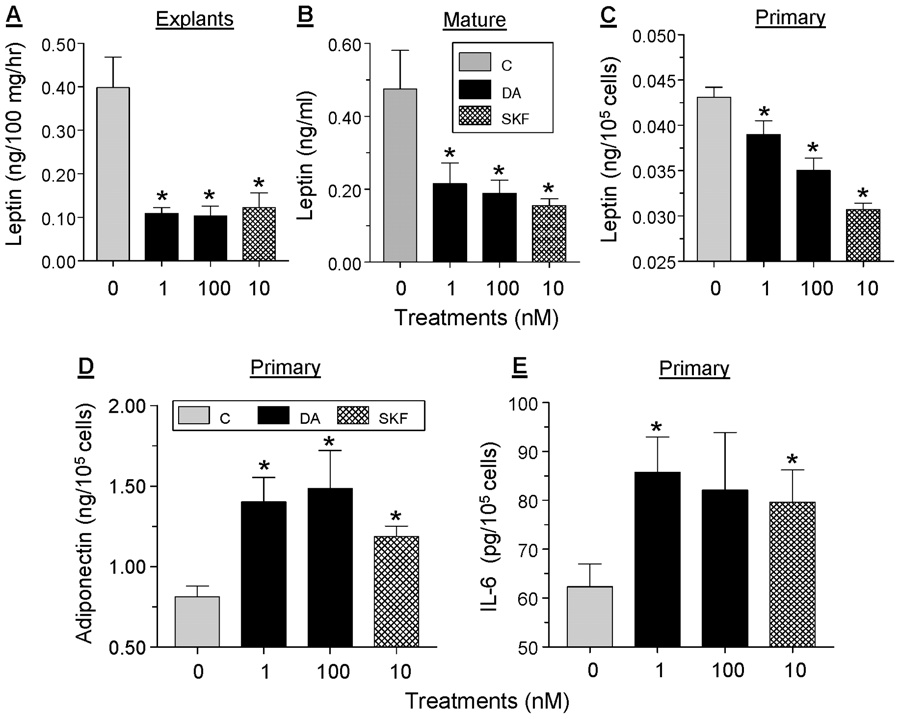
Effects of DA on adipokine/cytokine release. Sc explants (**panel A**), isolated mature adipocytes (**Panel B**) or differentiated primary adipocytes (**panel C**) were incubated with 1 or 100 nM DA or with 10 nM of SKF38393, a D1R/D5R agonist, for 24 hr. Leptin concentration in CM was determined by ELISA. Each value is a mean±SEM of 5 determinations; *, p<0.05. **D**, differentiated primary adipocytes were incubated as above and adiponectin release was determined by ELISA. **E**, proliferating primary adipocytes were incubated with 1 or 100 nM DA or with 10 nM of SKF for 6 hr and IL-6 release was determined by ELISA.

The effects of low (1 nM) and high (100 nM) doses of DA on leptin release were examined using sc adipose explants (**Fig. 6A**), isolated mature adipocytes (**Fig. 6B**), and differentiated primary adipocytes (**Fig. 6C**). Within 24 hr of incubation, leptin release was inhibited by 40–80% by either dose of DA. The potent D1R/D5R antagonist, SKF38393 at 10 nM, mimicked the inhibitory effect of DA, suggesting an action via D1R-like receptor while not excluding involvement of other receptors. Within 24 hr of incubation, both DA and SKF caused 60–80% stimulation of adiponectin release from differentiated primary adipocytes (**Fig. 6D**). Incubation of proliferating primary adipocytes for 6 hr with 1 nM DA or 10 nM SKF resulted in a moderate, 30–40% stimulation of IL-6 release (**Fig. 6E**).

## Discussion

This is the first report on expression of functional DAR in human adipose tissue and adipocytes. Our data suggest that D2R mediate the inhibitory effect of DA on adipocyte PRL gene expression and release, whereas D1R-like receptors are involved in the regulation of adipokine/cytokine release. A second important finding is that human adipose tissue and adipocytes express an active ARSA, capable of de-conjugating DA-S to bioactive DA. Collectively, these findings underscore a novel role for the peripheral dopaminergic system in the regulation of adipose tissue functions, with many implications to metabolic homeostasis in health and disease. For example, we propose that the ability of widely-prescribed antipsychotic medications, most of which act by binding to various DAR, to cause weight gain and alter metabolic homeostasis [34], [36], could be due, in part, to their direct action on adipose tissue.

The D1-like and D2-like receptors differ in sequence, ligand binding, coupling to Gs and Gi proteins, and signaling pathways [2], [7], [8], with peripheral DAR exhibiting different properties than their brain counterparts due to coupling to various effectors. Given the 80% sequence homology of D1R and D5R, they lack discriminating agonists and antagonists. Our Western blots showed evidence for several DAR isoforms in adipocytes, the ratio of which was altered following differentiation. Upon probing with an ab against D5R, multiple bands were seen (data not shown), which could be due either to D5R isoforms or to non-specificity of the ab. The difference in DAR expression between the SVC fraction and mature adipocytes (Fig. 1B) could be due to DAR expression by resident cells such as lymphocytes [37], [38] and endothelial cells [31] and should be further examined.

**Figure 7:**
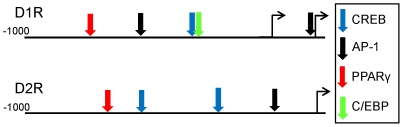
Putative transcription factor binding sites in the promoters of D1R and D2R. Sites were identified using Genomatix MatInspector. The diagram is not drawn to scale.

Both Western blotting and qPCR showed dissimilar changes in D1R and D2R expression during adipogenesis, with the most notable changes occurring during the first three days. Using the Genomatix MatInspector program, we identified several putative binding sites or transcription factors in the promoters of D1R and D2R. As illustrated in **Fig. 7**, both receptors have CREB and AP-1 binding sites which could respond to DAR activation of PKG/CREB and MAPK signaling. Notably, there is also a PPARγ binding site in both receptors, and a C/EBP binding site in D1R. The C/EBPs and PPARγ are sequentially expressed during early differentiation and are critical for the induction and maintenance of adipogenesis. The presence of their consensus binding sites in DAR promoters raises the possibility that similar to β-AR [39], [40], DAR are transcriptionally regulated during early adipogenesis by these factors.

The large concentration of DA-S in human serum is commonly overlooked because most of the methods for measuring CA do not detect DA-S. Sulfoconjugation is the major form of CA inactivation in human serum, while glucuronidation predominates in rats [16], [18]. In humans, a single amino acid substitution (Glu 146) in SULT1A3 sulfotransferase confers the enzyme with a higher affinity for DA than NE or Epi [41]. No orthologue of SULT1A3 has been found in rodents, suggesting a greater importance of DA sulfoconjugation in humans than in rodents [42].

Adipose tissue is rich in ARSC (steroid sulfatase) which de-conjugates sulfated steroids [43]. Together with aromatase, ARSC maintains high local estrogen levels in the breast, and has been studied for its impact on breast cancer [21]. Our data are the first to demonstrate active ARSA in human adipose tissue. The marked rise in enzyme activity after differentiation (**Fig. 2B**) could be due to increased enzyme expression, or an increase in co-factors such as saposin B [44] during adipogenesis. In addition to sulfated CA, ARSA uses galactosylceramide sulfate as a substrate. Inherited ARSA deficiency causes metachromatic leukodystrophy, a rare lysosomal storage disease whose manifestation ranges from lethality to motor and cognition deficits [45], [46]. Over 100 mutations in ARSA are known [47], with some causing total loss of enzyme activity while others resulting only in mild impairment in enzyme efficacy. Future studies should examine if polymorphisms in ARSA alter adipose tissue functions that are regulated by DA.

The control of extrapituitary PRL production is poorly understood. Studies by us [48] and others [49] showed no effects of DA on PRL release from human decidual explants. This was interpreted as insensitivity of the superdistal PRL promoter to DA rather than an absence of DAR in the decidua. The present data show that similar to the pituitary, DA suppresses adipocyte PRL gene expression and release via D2R. This likely occurs through inhibition of cAMP, followed by the suppression of PKA activity. Notably, DA showed a non-monotonic dose dependent inhibition of PRL in all adipocytes (**Figs. 3** **and** **4**). This suggests that activation of inhibitory DAR at the low DA doses is masked by activation of stimulatory DAR at the higher doses. Whether this occurs via increased cGMP and/or MAPK activation, remains to be determined. As depicted in **Fig. 4B**, the superdistal PRL promoter has several cAMP responsive elements such as CREB, and C/EBP, and two AP-1 sites which can respond to MAPK activation.

PRL has multiple roles in adipose tissue functions, including stimulating of adipogenesis, inhibition of lipolysis and variable effects on adipokine release (reviewed in [12]). Using visceral and sc explants from 50 patients, we found attenuated PRL release from sc explants from obese, but not in non-obese individuals [13]. Whether obesity is associated with alterations in ARSA activity, DAR expression, and/or changes in the signaling pathways that are activated by DA, is an intriguing issue which deserves further exploration. Notably, expression of PRL and PRLR showed an opposite pattern during early differentiation, followed by some recovery of the PRLR but a fall for PRL (**Fig. 1E**). Additional studies should examine the various factors that regulate expression of the above genes during adipogenesis.

Adipose tissue produces numerous adipokines and cytokines which participate in metabolic homeostasis and their dysregulation affects food consumption, body weight, lipid metabolism and inflammation [50], [51]. Whereas the release of leptin was strongly inhibited by DA, adiponectin and IL-6 were moderately stimulated, suggesting differential effects of the dopaminergic system on three important adipokines/cytokines. The ability of SKF, a D1R/D5R agonist, to mimic the actions of DA on adipokine release suggests involvement of D1R-like receptors. At present, neither of our human adipocyte cell line produces sufficient amounts of leptin or adiponectin to enable an in-depth mechanistic studies. As was recently reported for increased expression of leptin in 3T3-L1 adipocytes [52], we are experimenting with different culture conditions of LS14 and/or SW872 cells to resolve this issue. Our attempts to verify the role of MAPK in mediating the effects of DA on adipokine release were complicated by the fact that incubation of adipose explants or mature adipocytes with 5 µM U0126, a specific MEK inhibitor, markedly suppressed leptin release (data not shown). This suggested that another system(s), which is sensitive to MAPK inhibition, is involved in leptin release.

**Figure 8:**
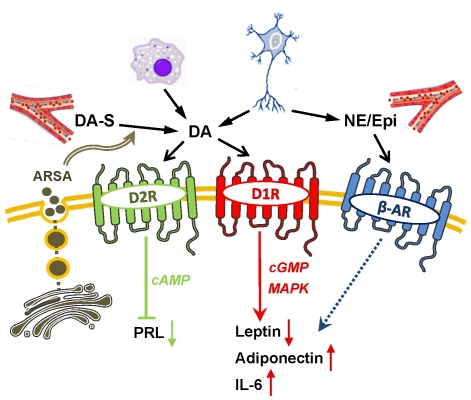
A model depicting the involvement of DA/DAR system in adipocyte functions. DA can reach the adipocytes from infiltrating lymphocyte/macrophages, sympathetic nerve endings or via the circulation in the form of DA-S. DA-S can become de-conjugated to bioactive DA by ARSA which is secreted from the lysosomes. DA binds to either D2R-like or D1R-like membrane receptors. Activation of D2R causes suppression of cAMP and inhibition of PRL gene expression and release. Activation of D1R-like receptors results in the inhibition of leptin release and stimulation of adiponectin and IL-6, an effect which may be mediated via the cGMP or MAPK signaling. Other catecholamines (i.e. NE and Epi) from the circulation or sympathetic neurons activate β-AR and modulate, by yet an unknown fashion, adipokine release. See text for additional explanations. ARSA, arylsulfatase A; β-AR, β-adrenergic receptors; D2R/D1R, type 1 or type 2 dopamine receptors; DA, dopamine; DA-S, dopamine sulfate; Epi, epinephrine; NE, norepinephrine; MAPK, mitogen-activated protein kinase; PRL, prolactin.

**Fig. 8** shows our proposed model on the involvement of the peripheral dopaminergic system in adipose tissue functions. Both DA-S and DA can reach the adipocytes via the circulation, from infiltrating lymphocytes/macrophages, and from local sympathetic nerve endings. Lysosomal ARSA is secreted into the adjacent extracellular space where it can de-conjugate DA-S, enabling binding of free DA to its receptors. It is unlikely that adipocytes convert DA to NE, as this requires not only DA internalization, but also expression of dopamine beta hydroxylase, which is presumably specific to secretory granules within neurons and adrenal chromaffin cells [1]. Activation of D2R by DA inhibits AC and suppresses intracellular cAMP. This leads to inactivation of PKA/CREB-responsive elements within the superdistal promoter that regulates PRL gene expression, followed by a reduction in PRL release. The actions of DA on leptin, adiponectin and IL-6 release are mediated by D1R-like receptors via signaling pathways that may include PKA, PKG, or MAPK. Our model also assume that β-AR, which are abundantly expressed in adipocytes [53] contribute, in an unclear manner, to adipokine/cytokine release.

Finally, dopaminergic altering drugs are prescribed to millions of patients with neuro-psychiatric disorders [54], [55]. Many of these drugs cause excessive weight gain, alter metabolic homeostasis and increase the risk of death from cardiovascular disease [56]. These effects have been solely attributed to the action of these drugs within the brain. The data reported here should inspire the reassessment of undesirable side effects of antipsychotics, by considering their ability to directly affect adipocyte functions that can lead to weight gain or changes in lipid metabolism and circulating adipokines.
